# Quantitative CT analysis for predicting the PD-L1 expression in lung adenocarcinoma

**DOI:** 10.1007/s11604-025-01857-8

**Published:** 2025-08-26

**Authors:** Masaya Tanabe, Yoshie Kunihiro, Masahiro Tanabe, Fumi Kameda, Masatoshi Nakashima, Taiga Kobayashi, Toshiki Tanaka, Yoshinobu Hoshii, Katsuyoshi Ito

**Affiliations:** 1 Present Address: Department of Radiology, Yamaguchi University Graduate School of Medicine, 1-1-1 Minamikogushi, Ube, Yamaguchi 755-8505 Japan; 2 Department of Radiology, National Hospital Organization Kanmon Medical Center, 1-1 Chofusotouracho, Shimonoseki, Yamaguchi 752-8510 Japan; 3 Department of Radiology, Ube Central Hospital, 750 Nishikiwa, Ube, Yamaguchi 755-0151 Japan; 4 Department of Surgery and Clinical Science, Division of Chest Surgery, Yamaguchi University Graduate School of Medicine, 1-1-1 Minamikogushi, Ube, Yamaguchi 755-8505 Japan; 5 Department of Diagnostic Pathology, Yamaguchi University Hospital, 1-1-1 Minamikogushi, Ube, Yamaguchi 755-8505 Japan

**Keywords:** X-ray computed tomography, Lung adenocarcinoma, Quantitative analysis, Multivariate analysis, Programmed death ligand 1

## Abstract

**Purpose:**

The objective of this study was to explore the relationship between a quantitative CT analysis and the expression of programmed death-ligand 1 (PD-L1) in lung adenocarcinoma.

**Materials and methods:**

This study included 116 patients diagnosed with lung adenocarcinoma who were assessed for the expression of PD-L1. Tumors were classified as pure ground-glass nodules (GGNs), part-solid nodules, and solid nodules. The quantitative CT analysis included the tumor diameter and volume, solid component diameter and volume, and rate of the solid components. The CT criteria, and PD-L1 expression rates were compared based on the tumor proportion score (TPS). Optimal cutoff values were obtained utilizing the maximized Youden index method based on the receiver operating characteristic (ROC) analysis. Univariate and multiple linear regression analyses were also performed to examine the influencing factors of 50% and 1% PD-L1 expression.

**Results:**

Solid nodules were significantly more frequent in the TPS ≥ 50% group (TPS ≥ 50% = 81.8% vs. TPS < 1% = 10.0%). The rate of solid component diameter and rate of solid component volume were significantly smaller in TPS < 1% than in TPS < 50% and 1–49% (p < 0.001, respectively). Multiple linear regression analysis identified the rate of solid component volume as a significant factor influencing 50% and 1% PD-L1 expression (p < 0.001 and p = 0.048, respectively).

**Conclusion:**

High PD-L1 expression rates may be associated with higher rates of solid components in lung adenocarcinoma.

## Introduction

Non-small-cell lung cancer (NSCLC) accounts for 80 to 85% of all primary lung cancers and is the leading cause of cancer-related death in many developed nations [1].

CT is essential for the diagnosis of lung cancer. Radiologic staging based on the 8th lung cancer TNM classification depends on the tumor size and the presence and size of the solid component [2]. Additionally, CT volumetry is useful for the quantitative analysis of lung nodules [3]. The CT workstation, a three-dimensional (3D) analysis system, enables artificial intelligence (AI)-driven measurement of the volume of the entire tumor, as well as the solid and ground-glass nodule (GGN) components.

Upon the diagnosis of NSCLC, genetic testing was performed to identify potential mutations associated with cancer cell proliferation. Most molecular-targeted drugs target and inhibit genetic mutations/translocations, referred to as drivers. The major driver genes included epidermal growth factor receptor (EGFR), anaplastic lymphoma kinase (ALK), c-ros oncogene 1 (ROS1), Kirsten rat sarcoma viral oncogene homolog (KRAS), B-Raf proto-oncogene serine/threonine-protein kinase (BRAF), and human epidermal growth factor receptor 2 (HER2). The rate of programmed death-ligand 1 (PD-L1) expression was also considered when deciding on a treatment plan. Immune checkpoint inhibitors targeting PD-L1 have demonstrated better progression-free and overall survival than conventional chemotherapy in patients with advanced NSCLC [4–6]. If the driver gene is negative, the rate of PD-L1 gene expression is assessed to determine whether immune checkpoint inhibitors can be expected to work. If the PD-L1 expression is > 50%, the patient should be treated with an immune checkpoint inhibitor (e.g., a PD-L1 inhibitor) alone or in combination with chemotherapy [7]. If the PD-L1 expression is 1–49%, immune checkpoint inhibitors and chemotherapy are used [8]. If the PD-L1 expression is < 1%, in addition to chemotherapy and PD-L1 inhibitors, treatment with a combination of two different immune checkpoint inhibitors, a PD-L1 inhibitor and a cytotoxic T-lymphocyte antigen-4 (CTL-A4) inhibitor, may be an option [9].

There have been several reports on the relationship between the PD-L1 expression and CT imaging findings [10–16]. However, the relationship between the parameters of a quantitative CT analysis and the expression of PD-L1 in pT1 lung adenocarcinoma has not been well evaluated. It could be useful to predict the PD-L1 expression in patients who undergo neoadjuvant immunotherapy.

The objective of this study was to explore the relationship between quantitative CT analysis and the expression of PD-L1 in pT1 lung adenocarcinoma.

## Materials and methods

This study was approved by the institutional review board of our hospital. The requirement for informed consent was waived due to its retrospective design.

### Study population

**Fig. 1:**
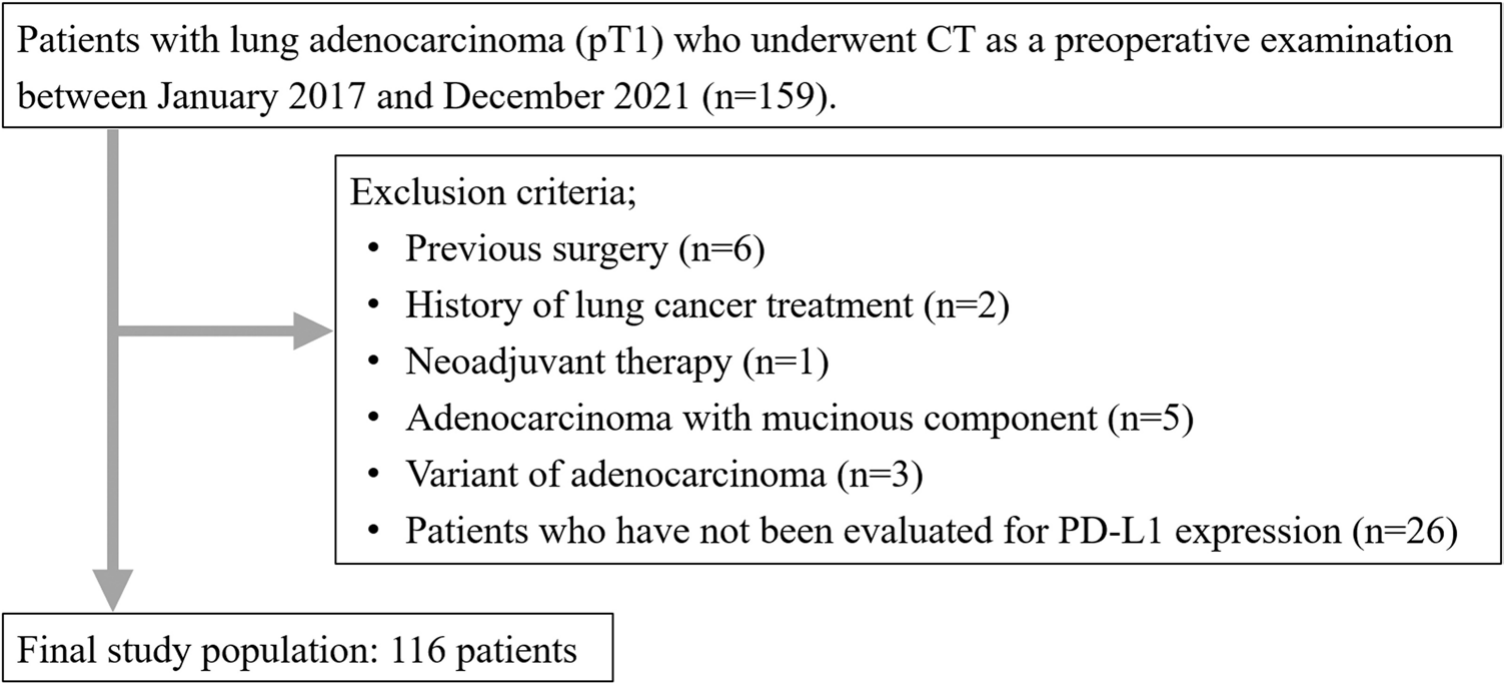
Flowchart of the patient selection

This study included patients who underwent preoperative CT for lung cancer between January 2017 and December 2021. The inclusion criteria were patients who underwent CT imaging within two months before surgery and who were pathologically diagnosed with pT1 lung adenocarcinoma. During the study period, 159 patients met the inclusion criteria. Patients with history of previous lung operation (n = 6), lung cancer treatment (n = 2), neoadjuvant therapy (n = 1), adenocarcinoma with mucinous component (n = 5), variants of adenocarcinoma or other accompanying tumor components (n = 3; two cases with adenosquamous carcinoma and one case with adenocarcinoma with neuroendocrine feature), and patients in whom the expression of PD-L1 was not evaluated (n = 26) were excluded from the study (Fig. 1).

Thus, the final study population consisted of 116 patients (male, n = 65; female, n = 51; mean age: 71.6 ± 9.0 [range: 37–85 years]) (Table 1). Smoking history was identified in 69 patients (59.5%; mean pack years: 38.7 ± 24.5). The diagnoses of lung adenocarcinoma included minimally invasive adenocarcinoma (MIA) (n = 15) and invasive adenocarcinoma (IAC) (n = 101). The analysis was further stratified based on the PD-L1 expression status. The PD-L1 expression status was classified based on the tumor proportion score (TPS) [17]. Eleven (9.5%) patients had TPS ≥ 50%, 45 (38.8%) patients had TPS 1–49%, and 60 (51.7%) patients had TPS < 1%. Pathological stages were determined based on the International Union Against Cancer 8 definitions.

**Table 1:** The clinical characteristics of patients with lung adenocarcinoma

	Lung adenocarcinoma (n = 116)
Age (years)*	71.6 ± 9.0
Sex, male/female	65/51 (56.0/44.0)
Smoking status	
Never	47 (40.5)
Former or current	69 (59.5)
Pathological diagnosis	
MIA	15 (12.9)
IAC	101 (87.1)
Pathological stage	
I	107 (92.2)
II	7 (6.0)
III	2 (1.7)
PD-L1 expression	
TPS: ≥ 50%	11 (9.5)
TPS: 1–49%	45 (38.8)
TPS: < 1%	60 (51.7)

Unless otherwise specified, data are numbers of patients, with percentages in parentheses

^*^Data are means ± standard deviations

*MIA* minimally invasive adenocarcinoma, *IAC* invasive adenocarcinoma, *PD-L1* programmed death-ligand 1, *TPS* tumor proportion score

### CT technique

Multidetector-row CT (MDCT) examinations were performed using SOMATOM Force or Drive (Siemens Healthineers, Erlangen, Germany), or Aquilion Precision (Canon Medical Systems, Otawara, Japan). All scans were performed in a cephalocaudal direction. The imaging parameters for thin-section CT were as follows: tube voltage, 100 kVp; scan field of view, 320–360 mm; slice thickness, 1 mm. The tube current was determined using an automatic exposure-control system. CT images for lung windows were reconstructed using kernel Br64 and ADMIRE strength 1 for the Siemens CT systems and using kernel FC53 and AIDR 3D weak for the Canon CT systems. Monitors were used to view the CT image at lung settings (window width 1500 HU and window level, −600 HU).

### CT image analysis

Two radiologists (M. N., with 1 year of experience and F. K., with 10 years of experience) classified the morphology of tumors into pure GGN, part-solid nodules, and solid nodules. Differences were resolved by consensus. The longest diameters of the overall tumor of the lung cancer (tumor diameter), including the solid and GGN components, and the longest diameters of the solid component (solid component diameter) were measured on any one of axial, sagittal, and coronal CT images by the two radiologists. The averages of the data were used in the analysis. From these CT measurements, the solid component diameter (%) was calculated as follows: solid component diameter/tumor diameter × 100 (%). All CT datasets were transferred to a dedicated lung application for the lungs (Volume Analyzer; SYNAPSE VINCENT, Fujifilm Corp, Tokyo, Japan). The tumor was dragged by one radiologist (F.K.) and the volume data including the tumor volume and the solid component volume were automatically analyzed. The results were confirmed by the radiologist (F.K.). The rate of the solid component volume was calculated as follows: solid component volume/tumor volume × 100 (%).

### Statistical analysis

Data were analyzed using SPSS (ver. 27.0, IBM, Armonk, NY, USA). Continuous variables are presented as means ± standard deviations or medians with interquartile ranges (IQRs). To compare tumor morphology between the groups of TPS ≥ 50%, 1–49% and < 1%, we used Fisher’s exact probability test. An adjusted standardized residual of > 1.96 or <  −1.96 was considered indicative of a group with a significantly higher or lower frequency, respectively. The Kruskal–Wallis test was used to compare the age, smoking history and CT measurements between the groups of TPS ≥ 50%, 1–49% and < 1%. If the Kruskal–Wallis test showed a statistically significant *p*-value, the Dunn-Bonferroni post-hoc method for all pairwise comparisons were performed. Additionally, receiver operating characteristic (ROC) curves were plotted and the areas under the curve (AUC) were calculated. Optimal cutoff values were obtained utilizing the maximized Youden index method based on the ROC analysis. Univariate and multiple linear regression analyses were also performed to examine the influencing factors of 50% and 1% PD-L1 expression. Statistical significance was set at *p* < 0.05.

Interobserver agreement between the two radiologists was calculated as the kappa value (κ) for the tumor morphology and as the intraclass correlation coefficient (ICC) for the tumor diameter and the solid component diameter as follows: slight (0.00–0.20), fair (0.21–0.40), moderate (0.41–0.60), substantial (0.61–0.80), or almost perfect (0.81–1.00).

## Results

### CT analysis

**Fig. 2:**
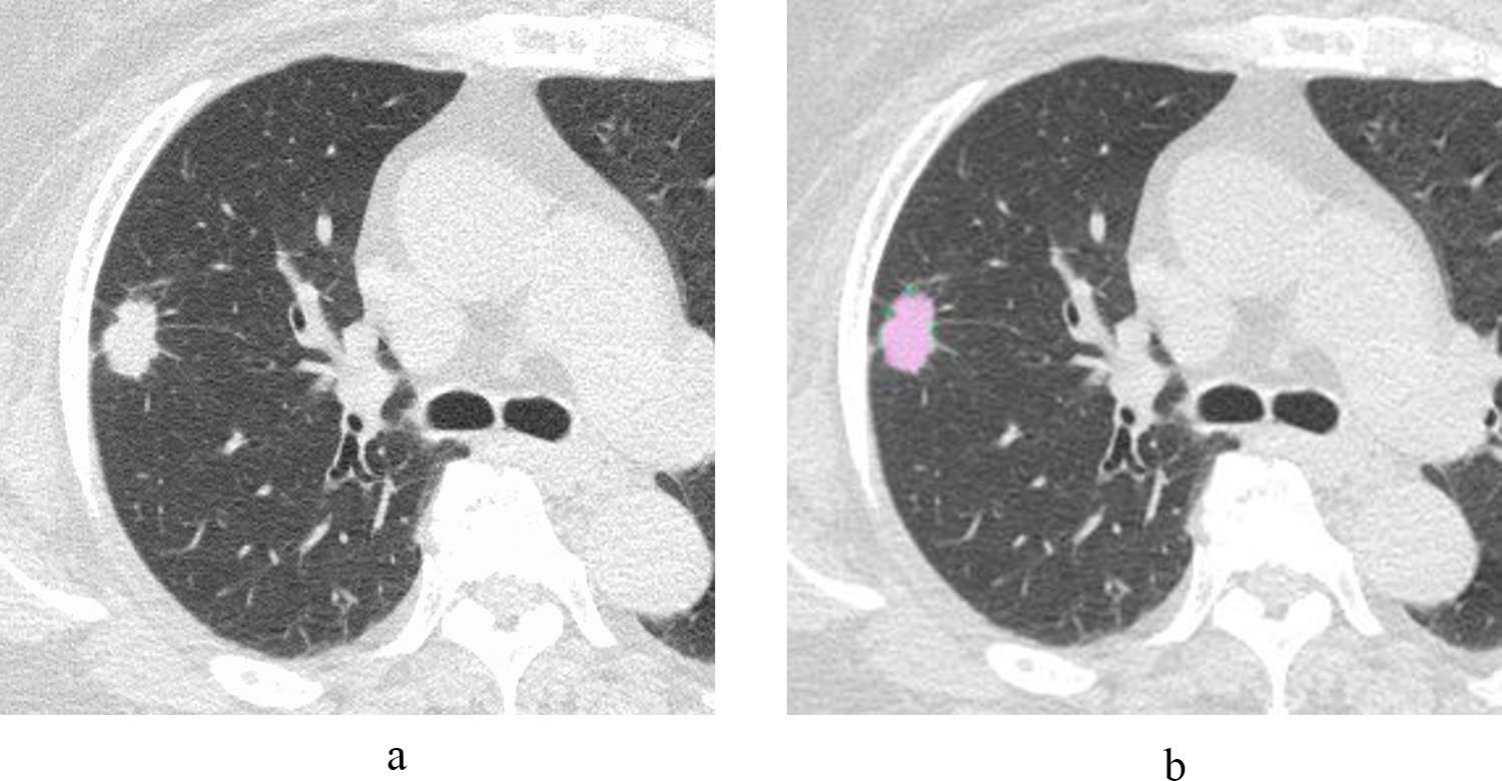
A 68-year-old woman with invasive adenocarcinoma (TPS ≥ 50%). **a** HRCT shows a solid nodule. The diameters of both the tumor and solid component are 17.7 mm. The rate of the solid component diameter is 100%, **b** The volume of the tumor and solid component are 2206.8 mm^3^ (pink and green area) and 2195.1 mm^3^ (pink area), respectively. The rate of the solid component volume is 99.5%. *TPS* tumor proportion score, *HRCT* high-resolution CT

**Fig. 3:**
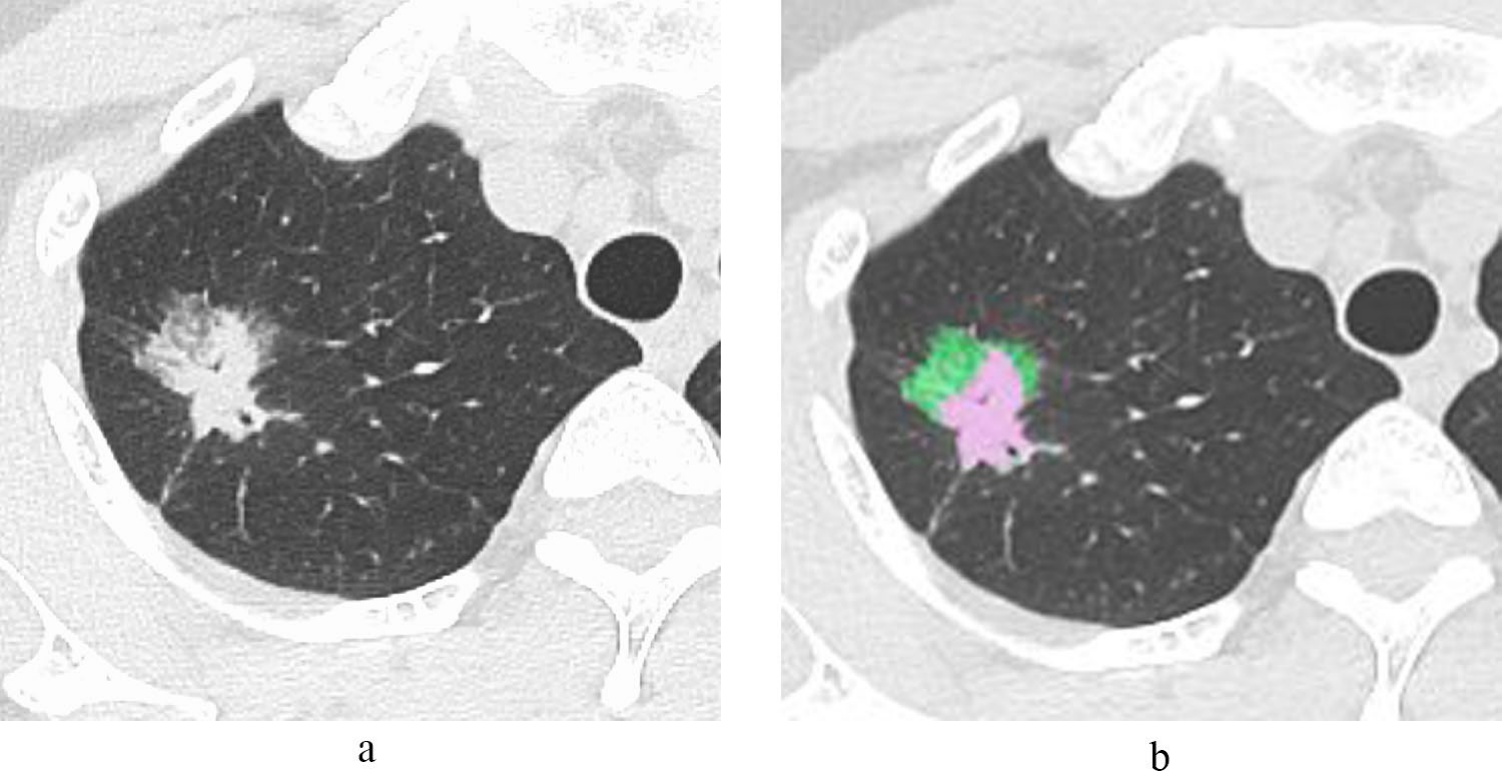
A 46-year-old woman with invasive adenocarcinoma (TPS = 0%). **a** HRCT shows a part-solid nodule. The diameters of the tumor and solid component are 28.7 mm and 19.1 mm, respectively. The rate of the solid component diameter is 66.6%, **b** The volume of the tumor and solid component are 5752.3 (pink and green area) mm^3^ and 3301.6 mm^3^ (pink area), respectively. The rate of the solid component is 57.4%. *TPS* tumor proportion score, *HRCT* high-resolution CT

Table 2 shows the results of the CT analysis between the groups of TPS ≥ 50%, 1–49% and < 1%. A significant difference in tumor morphology was seen between the groups of TPS ≥ 50%, 1–49% and < 1% (*p* < 0.001). Part-solid nodules were significantly more frequent in the TPS < 1% group (TPS ≥ 50% = 18.2% vs. TPS < 1% = 81.7%), whereas solid nodules were significantly more frequent in the TPS ≥ 50% group (TPS ≥ 50% = 81.8% vs. TPS < 1% = 10.0%). No significant differences were observed in age, tumor diameter, solid component diameter, tumor volume and solid component volume between the groups of TPS ≥ 50%, 1–49% and < 1%. The rate of solid component diameter and rate of solid component volume were significantly smaller in TPS < 1% than in TPS < 50% and 1–49% (TPS ≥ 50%, 1–49% and < 1%; median rate of solid component diameter: 100%, 86.5% and 60.7%, *p* < 0.001; median rate of solid component volume: solid component diameter: 96.1%, 52.1% and 28.2%, *p* < 0.001, respectively) (Figs. 2, 3).

**Table 2:** Comparison between the groups of TPS ≥ 50%, 1–49% and < 1% in the CT analysis

	All (n = 116)	TPS ≥ 50% (n = 11)	TPS: 1–49% (n = 45)	TPS < 1% (n = 60)	*p* value
Tumor morphology^†^					
Pure GGN	6 (5.2)	0	1 (2.2)	5 (8.3)	< 0.001
Part-solid nodule	79 (68.1)	2 (18.2)^*^	28 (62.2)	49 (81.7)^**^	
Solid nodule	31 (26.7)	9 (81.8)^**^	16 (35.6)	6 (10.0)^*^	
Age [years]^‡^	71.6 ± 9.0	74.6 ± 5.0	73.3 ± 6.5	69.8 ± 10.8	0.258
Smoking history [pack years]	14.3 (40)	20.0 (32.1)	28.5 (45.8)	1.25 (30.8)	0.030^§^
Tumor diameter [mm]	20.9 (12.5)	18.4 (8.2)	20.3 (11.3)	24.2 (14.6)	0.245
Solid component diameter [mm]	14.5 (11.9)	16.4 (3.7)	15.0 (10.9)	10.7 (15.2)	0.100
Rate of solid component diameter [%]	68.7 (51.9)	100 (10.9)	86.5 (47.8)	60.7 (49.8)	< 0.001^||^
Tumor volume [mm^3^]	2248.3 (3964.3)	1650.0 (1533.2)	2472.1 (3486.2)	2649.6 (4635.7)	0.538
Solid component volume [mm^3^]	939.5 (1805.7)	1642.1 (1315.7)	1006.2 (1891.0)	585.2 (1859.4)	0.109
Rate of solid component volume [%]	40.6 (57.9)	96.1 (22.4)	52.1 (57.4)	28.2 (37.9)	< 0.001^¶^

Unless otherwise specified, data are the median, with the interquartile range in parentheses

^†^Data are numbers of patients, with percentages in parentheses

^‡^Data are means ± standard deviations

^*^Significantly lower (adjusted standard residuals < −1.96) in the groups

^**^Significantly higher (adjusted standard residuals > 1.96) in groups

^§^Post-hoc analysis: TPS ≥ 50% vs 1–49%; *p* = 1.000, TPS ≥ 50% vs < 1%; *p* = 0.216, TPS 1–49% vs < 1%; *p* = 0.058

^||^Post-hoc analysis: TPS ≥ 50% vs 1–49%; *p* = 0.113, TPS ≥ 50% vs < 1%; *p* < 0.001, TPS 1–49% vs < 1%; *p* = 0.002

^¶^Post-hoc analysis: TPS ≥ 50% vs 1–49%; *p* < 0.016, TPS ≥ 50% vs < 1%; *p* < 0.001, TPS 1–49% vs < 1%; *p* = 0.004

*TPS* tumor proportion score, *GGN* ground-glass nodule

Interobserver agreement was substantial to almost perfect (κ = 0.64 for the tumor morphology, ICC = 0.81 for the tumor diameter, and ICC = 0.74 for the solid component diameter).

### ROC curve analysis

**Fig. 4:**
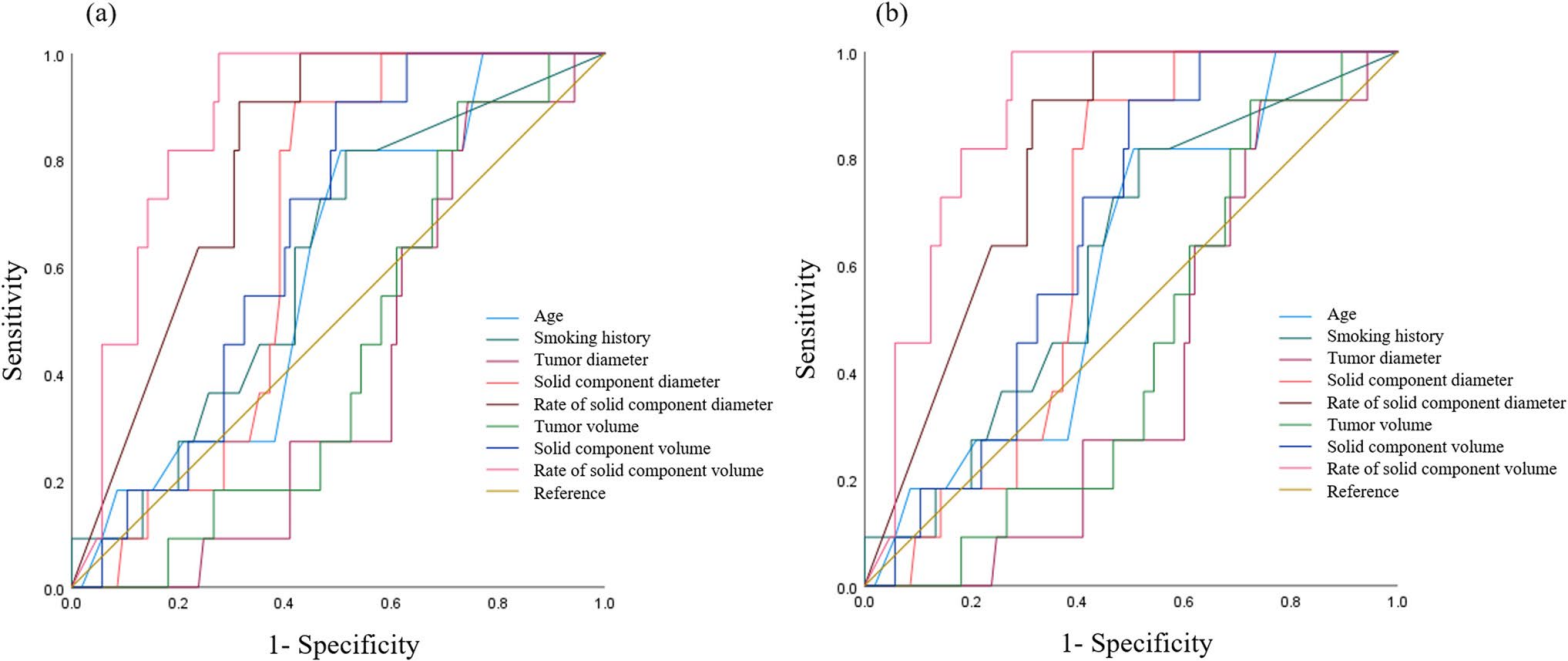
ROC curves predicting PD-L1 expression thresholds of 50% (**a**) and 1% (**b**). *ROC* receiver operating characteristic, *PD-L1* programmed death-ligand 1

ROC curve analysis was performed to investigate the predicting factor for 50% and 1% PD-L1 expression in the age, smoking history and CT measurements (Fig. 4). Table 3 shows the AUC, optimal cutoff, sensitivity, and specificity for PD-L1 expression thresholds of 50% and 1%, respectively. The AUC values for the PL-L1 50% threshold and PD-L1 1% threshold were 0.403–0.876 and 0.415–0.735, respectively.

**Table 3:** AUC, optimal cutoff, sensitivity, and specificity for PD-L1 expression thresholds of 50% and 1%

	AUC (95%CI)	Optimal cutoff	Sensitivity	Specificity
PD-L1 50% threshold				
Age [years]	0.600 (0.451–0.748)	72.5	0.82	0.50
Smoking history [pack years]	0.610 (0.453–0.766)	12.4	0.82	0.49
Tumor diameter [mm]	0.403 (0.273–0.532)	15.3	0.91	0.26
Solid component diameter [mm]	0.656 (0.548–0.764)	15.3	0.91	0.58
Rate of solid component diameter [%]	0.801 (0.708–0.895)	86.6	0.91	0.69
Tumor volume [mm^3^]	0.441 (0.305–0.576)	1035.1	0.91	0.28
Solid component volume [mm^3^]	0.664 (0.544–0.784)	803.8	0.91	0.55
Rate of solid component volume [%]	0.876 (0.806–0.945)	60.0	1.00	0.72
PD-L1 1% threshold				
Age [years]	0.582 (0.478–0.686)	65.5	0.95	0.25
Smoking history [pack years]	0.636 (0.534–0.738)	12.4	0.70	0.60
Tumor diameter [mm]	0.415 (0.310–0.520)	14.4	0.80	0.22
Solid component diameter [mm]	0.597 (0.492–0.703)	10.9	0.79	0.52
Rate of solid component diameter [%]	0.716 (0.621–0.810)	85.8	0.61	0.82
Tumor volume [mm^3^]	0.442 (0.337–0.548)	9144.0	0.14	0.90
Solid component volume [mm^3^]	0.589 (0.485–0.693)	456.5	0.79	0.43
Rate of solid component volume [%]	0.735 (0.643–0.827)	66.7	0.52	0.90

*AUC* area under the curve, *PD-L1* programmed death-ligand 1, *CI* confidence interval

### Multiple linear regression analyses

Table 4 details the impact of age, smoking history, Tumor diameter, solid component diameter, rate of solid component diameter, tumor volume, solid component volume, and rate of solid component volume. Multiple linear regression analysis identified the rate of solid component volume as a significant factor influencing 50% and 1% PD-L1 expression (*p* < 0.001 and *p* = 0.048, respectively).

**Table 4:** Multiple linear regression analyses about influencing factors of 50% and 1% PD-L1 expression

	Univariate analysis			Multivariate analysis		
	B	SE	*p*	B	SE	*p*
PD-L1 50% threshold						
Age [years]	0.004	0.003	0.246			
Smoking history [pack years]	0.001	0.001	0.299			
Tumor diameter [mm]	−0.004	0.003	0.213			
Solid component diameter [mm]	0.003	0.003	0.302			
Rate of solid component diameter [%]	0.003	0.001	0.002^*^	−0.001	0.001	0.561
Tumor volume [mm^3^]	−0.000006317	0.000	0.234			
Solid component volume [mm^3^]	0.00000531	0.000	0.640			
Rate of solid component volume [%]	0.004	0.001	< 0.001^*^	0.004	0.001	< 0.001^*^
PD-L1 1% threshold						
Age [years]	0.011	0.005	0.027^*^	0.008	0.005	0.106
Smoking history [pack years]	0.004	0.002	0.011^*^	0.003	0.002	0.055
Tumor diameter [mm]	−0.008	0.005	0.105			
Solid component diameter [mm]	0.007	0.005	0.170			
Rate of solid component diameter [%]	0.006	0.001	< 0.001^*^	0.002	0.002	0.324
Tumor volume [mm^3^]	−0.000004071	0.000	0.654			
Solid component volume [mm^3^]	0.000009645	0.000	0.618			
Rate of solid component volume [%]	0.006	0.001	< 0.001^*^	0.004	0.002	0.048^*^

^*^Significant statistical difference (*p* < 0.05)

*B* partial regression coefficient, *SE* standard error, *p p*-value

## Discussion

We investigated the ability of CT images to predict the PD-L1 expression in pT1 lung adenocarcinomas. There have been several reports on the relationship between the expression of PD-L1 and CT imaging findings, including advanced stages [10–16], however, our study which focused on pT1 cases and used evaluation by radiologists and a CT software analysis, showed that the rate of the solid component volume could be an indicator for predicting the expression of PD-L1.

Our study showed that solid nodules were significantly more frequent in TPS ≥ 50% group and part-solid nodules were significantly more frequent in TPS < 1% group. The results of our study support those of a previous study, which reported that PD-L1 expression was significantly associated with radiologic/pathologic invasive adenocarcinoma [15]. In this study, the number of patients with pure GGN was relatively small because pure GGN tends to not be resected immediately and recommended regular follow-up. No significant difference in CT patterns was reported in previous studies [10, 11]. However, the patients with advanced lung adenocarcinoma were evaluated and the number part-solid nodules was small in a previous study [10] and it is possible that the difference of the background factors effected the results of our study and the previous studies.

In our study, the tumor diameter and the tumor volume were not significantly different between the PD-L1-positive and PD-L1-negative cases. These were in line with the results of previous studies [10, 11]. In our study, the solid component diameter measured by radiologists and tumor volume measured using the CT software program were not associated with the expression of PD-L1, but a larger rate of solid component diameter measured by radiologists, larger rate of the solid component volume measured using the CT software program were associated with the expression of PD-L1.

A solid component on CT tends to indicate an invasive lesion in lung adenocarcinoma and is related to worse prognosis [2, 18]. The solid component could correspond to stromal or vascular invasion, collapsed alveolar space, fibroblastic proliferation, infiltration, and mucin [19]. Our study also revealed that solid component in non-mucinous lung adenocarcinoma could be an indicator for PD-L1 expression and useful for treatment selection.

Three-dimensional analysis systems, such as the SYNAPSE VINCENT workstation, have been able to extract highly accurate 3D images from CT and analyze them using AI with deep learning [20–22]. In SYNAPSE VINCENT’s lung nodule extraction program, the AI not only automatically extracts lung nodules from CT images of lung fields but also measures the tumor diameter and volume, and even divides the tumor into solid components and GGN. While conventional methods of manual measurement by humans usually yield inconsistent measurement results, the use of automatic measurement by AI has made it possible to obtain consistent results, irrespective of the timing of measurement.

Recently, several studies have investigated the relationship between positron emission tomography (PET)/CT and the expression of PD-L1 [23–26]. It is important to be able to predict the expression of PD-L1 using multiple modalities is necessary.

The present study was associated with several limitations. First, the study was retrospective in nature and was conducted at a single institution. Accordingly, the number of patients with pathologically confirmed lung cancers was relatively small. Furthermore, some patients with a diagnosis of lung cancer were excluded because they had not undergone genetic testing for PD-L1. The relatively small study population limited the power of the statistical analysis. Second, this study focused on pT1 lung cancer cases and did not examine unresectable lung cancers. Further studies should analyze cases of unresectable lung cancer because immune checkpoint inhibitors are more commonly administered to patients with unresectable lung cancer. However, it is also necessary to predict the PD-L1 expression in patients who undergo neoadjuvant immunotherapy [27].

In conclusion, a quantitative CT analysis may be useful for predicting the expression of PD-L1 in lung adenocarcinoma. The rate of the solid component volume may be an indicator of the expression of PD-L1 in lung adenocarcinoma.
